# Signaling Pathways Related to Oxidative Stress in Diabetic Cardiomyopathy

**DOI:** 10.3389/fendo.2022.907757

**Published:** 2022-06-15

**Authors:** Meng-ling Peng, Yu Fu, Chu-wen Wu, Ying Zhang, Hang Ren, Shan-shan Zhou

**Affiliations:** ^1^ Department of Cardiology, The First Hospital of Jilin University, Changchun, China; ^2^ Department of Cardiology, The Second Hospital of Jilin University, Changchun, China

**Keywords:** diabetic cardiomyopathy, oxidative stress, signal pathway, inflammation, cardiac remodeling

## Abstract

Diabetes is a chronic metabolic disease that is increasing in prevalence and causes many complications. Diabetic cardiomyopathy (DCM) is a complication of diabetes that is associated with high mortality, but it is not well defined. Nevertheless, it is generally accepted that DCM refers to a clinical disease that occurs in patients with diabetes and involves ventricular dysfunction, in the absence of other cardiovascular diseases, such as coronary atherosclerotic heart disease, hypertension, or valvular heart disease. However, it is currently uncertain whether the pathogenesis of DCM is directly attributable to metabolic dysfunction or secondary to diabetic microangiopathy. Oxidative stress (OS) is considered to be a key component of its pathogenesis. The production of reactive oxygen species (ROS) in cardiomyocytes is a vicious circle, resulting in further production of ROS, mitochondrial DNA damage, lipid peroxidation, and the post-translational modification of proteins, as well as inflammation, cardiac hypertrophy and fibrosis, ultimately leading to cell death and cardiac dysfunction. ROS have been shown to affect various signaling pathways involved in the development of DCM. For instance, OS causes metabolic disorders by affecting the regulation of PPARα, AMPK/mTOR, and SIRT3/FOXO3a. Furthermore, OS participates in inflammation mediated by the NF-κB pathway, NLRP3 inflammasome, and the TLR4 pathway. OS also promotes TGF-β-, Rho-ROCK-, and Notch-mediated cardiac remodeling, and is involved in the regulation of calcium homeostasis, which impairs ATP production and causes ROS overproduction. In this review, we summarize the signaling pathways that link OS to DCM, with the intention of identifying appropriate targets and new antioxidant therapies for DCM.

## 1 Introduction

Diabetes mellitus (DM) is a serious metabolic disease. Its global prevalence among those aged 20 to 79 years old in 2021 was estimated to be 10.5% (536.6 million people), rising to 12.2% (783.2 million) in 2045 (1). Notably, DM is an independent risk factor for heart failure (HF). In the Framingham study, men aged 45 to 74 years had more than a two-fold higher risk of congestive failure than their nondiabetic cohorts, and diabetic women had a five-fold higher risk (2).

Diabetic cardiomyopathy (DCM) is characterized by gradually progressing HF symptoms and deleterious cardiac remodeling, leading to fibrosis and diastolic and systolic dysfunction, in the absence of coronary artery disease and hypertension. However, DCM is still not well defined, despite having been first identified 60 years ago. The most typical pathogenic features of DCM are glucose and lipid metabolic disorders, the glycation of proteins, oxidative stress (OS), inflammation, cardiac remodeling, and cardiac dysfunction (3). Notably, no specific symptoms of early stage DCM and no specific treatment for DCM patients have been reported. Therefore, early identification of DCM patients, delaying the progression of DCM, and finding targeted therapy strategies are urgently needed for DCM patients.

OS is considered to have an important role in the development of DCM. In diabetic environments, a variety of antioxidant enzymes are inactivated or show reductions in their activity, and the imbalance between the production of ROS and their elimination by oxidative defense systems is referred to as OS. Indeed, both ROS and reactive nitrogen species (RNS) are involved in OS, which damages DNA, proteins, mitochondria, the endoplasmic reticulum, and glucose and lipid metabolism through different signaling pathways, such as the PI3K/AKT, PPARα pathway (4).

Therefore, strategies designed to combat OS in diabetic patients could help develop new therapies for DCM. In this review, we focus on the molecular mechanisms of DCM and comprehensively analyze the signaling pathways related to OS in DCM. In this way, we have identified areas of uncertainty in this field that merit further study.

## 2 Major Pathogenic Features

### 2.1 Abnormalities in Glucose and Lipid Metabolism

**Figure 1:**
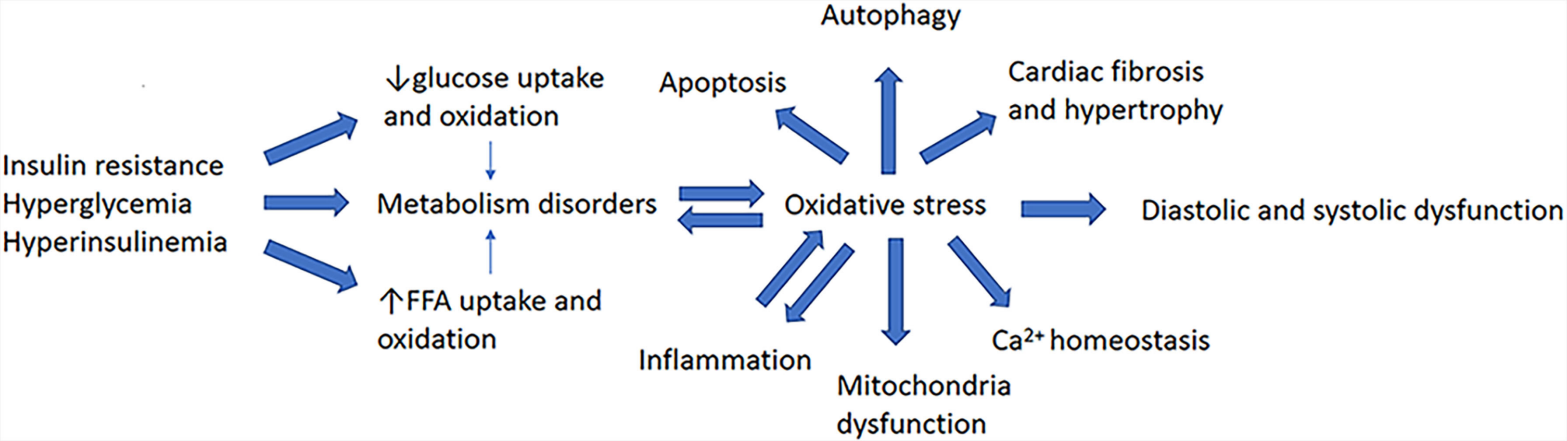
Major pathogenetic features of diabetic cardiomyopathy.

Abnormalities in glucose and lipid metabolism are the basic pathological features of DCM and a cause of OS in cardiomyocytes (**Figure 1**). Under normal circumstances, cardiomyocytes mainly depend on fatty acids (FAs) for energy, with only ~40% being derived from glucose. Greater FA uptake and lower glucose oxidation have been identified in the hearts of both patients and animal models with type 1 or type 2 diabetes (T1DM or T2DM) (5, 6) and DCM (D1CM or D2CM, respectively). Thus, there tends to be a switch in energy substrate utilization in the diabetic heart (7). Mechanistically, in insulin resistance (IR), hyperglycemia, and hyperinsulinemia, peroxisome proliferator-activated receptor α (PPARα) is activated, which increases the translocation of cluster of differentiation 36 (CD36) to the sarcolemma, where it promotes the uptake of FAs by cardiomyocytes (8). In addition, impairments in insulin signaling cause a reduction in the translocation of glucose transporter 4 (GLUT4), which is the principal mediator of insulin-stimulated glucose uptake. These defects contribute to greater FFA uptake and oxidation, and lower glucose uptake and oxidation. Of note, despite the elevated levels of FFA oxidation, the excess uptake of FFA is not fully oxidized. Cardiotoxic lipid intermediates, such as ceramide, accumulate because of the consequent imbalance between FFA uptake and oxidation, resulting in lipotoxicity and accumulation of ROS and RNS (9, 10). Excessive FFA oxidation is associated with an increase in the consumption of ATP by metabolic intermediates and a reduction in the efficiency of ATP production (11, 12). Thus, lower ATP production also contributes to cardiac dysfunction (13).

### 2.2 Glycated Proteins/O-Glycosylation

The accumulation of advanced glycation end-products (AGEs) and greater activation of the hexosamine biosynthesis pathway (HBP) have been shown to make an important contribution to mitochondrial damage induced by high glucose concentrations. AGEs are produced non-enzymatically from glycosylation reactions between glucose and protein or lipids, and are involved in the pathogenesis of DCM (14). The binding of AGEs to the receptor for AGEs (RAGEs) activates the nuclear factor (NF)-κB signaling pathway, which causes inflammatory cells to release a series of proinflammatory cytokines, chemokines, and exosomes. In addition, the functions of the proteins are affected. Finally, the deposition of AGEs in the extracellular matrix (ECM) and crosslinking with ECM proteins, impair the degradation of the ECM by matrix metalloproteinases (MMPs), which increases cardiac stiffness, and these effects together manifest in the form of early diastolic dysfunction (15).

The association between OS and DCM has also been demonstrated in animal models of diabetes, which show high *O*-linked N-acetylglucosamine (*O*-GlcNAc) concentrations in DCM. The presence of an excessive amount of glucose increases the number of *O*-GlcNAcylation events, which increases the post-translational modification of proteins in the diabetic heart, thereby modifying their activity, and further impairing mitochondrial function and ATP production (9, 16). An impairment in diabetes-induced relaxation is also linked to greater HBP activation, and influences cytosolic Ca^2+^ concentration (17) *via* a reduction in sarcoplasmic reticulum calcium-ATPase (SERCA) activity and slower Ca^2+^ reuptake (18). In addition, the O-GlcNAcylation of several mitochondrial respiratory complexes is increased, which inhibits their activation (19, 20). Finally, greater O-GlcNAcylation of proteins involved in autophagy, including Beclin-1, ULK-1, and mTOR, affects their activity (21). It has been reported that selective targeting of cardiac protein O-GlcNAcylation to restore physiological O-GlcNAc balance may represent a novel therapeutic approach for diabetes-induced heart failure (22).

### 2.3 Oxidative Stress

Under physiological conditions, mitochondria generate abundant ATP through oxidative phosphorylation in cardiomyocytes. They efficiently metabolize pyruvate and FFAs to generate acetyl-CoA, and the electron reduction process in the respiratory chain reduces a small amount of oxygen to ROS. This process requires enzymes, including NADPH oxidase (NOX), xanthine oxidase (XO), and monoamine oxidase (MAO). NOX2 is located in the cell membrane of cardiomyocytes and is a heterodimer, composed of NOX2 and P22^Phox^ subunits, that is activated alongside P47^phox^, p67^phox^, and Rac1, causing the release of O• directly into phagosomes or the extracellular space. However, hyperglycemia leads to the activation of NOX and XO, causing NO synthase (NOS) uncoupling, which is linked with cardiac remodeling in diabetes. NOX2 and NOX4 expression is upregulated while ROS-scavenging enzyme expression is downregulated in the heart of diabetic mice (23). ROS comprise a group of substances with strong oxidizing ability, such as superoxide and hydrogen peroxide, which can react with NO to form the peroxynitrite anion (ONOO^−^), which is directly cytotoxic and reduces NO bioavailability, further predisposing toward inflammation, mitochondrial dysfunction, and a progressive profibrotic response that induces ECM remodeling and fibrosis (6).

### 2.4 Inflammation

Inflammation plays an important role in the pathogenesis of cardiac dysfunction by causing an impairment in insulin signaling and reducing endogenous antioxidant concentrations. Indeed, there are numerous interactions between OS and inflammation. In DCM, the excess production of ROS provides a strong proinflammatory signal and causes myocardial tissue damage. In addition, the hyperglycemia associated with T2DM leads to greater mitochondrial respiration in endothelial cells (ECs), which increases ROS production and OS. A number of proinflammatory cytokines, such as IL-6, IL-18, and transforming growth factor β (TGF-β), and nucleotide-binding oligomerization domain-like receptor proteins (NLRPs), have been shown to be important in the inflammation that characterizes DCM (24).

The NLRP3 inflammasome is activated by overproduction of ROS, thereby increasing inflammatory damage. The NLRP3 inflammasome consists of NLRP3, apoptosis-associated speck-like protein (ASC), and pro-cysteinyl aspartate specific proteinase-1 (pro-caspase-1). It has been shown to interact with thioredoxin (TRX) binding protein-2 (TBP-2), and a high concentration of intracellular ROS causes the dissociation of TBP-2 from TRX, which can then bind to NLRP3 and activate it. Upon activation by ROS, NLRP3 recruits ASC and pro-caspase-1, leading to the activation of caspase-1, IL-1β, and IL-18 by proteolytic cleavage (25).

### 2.5 Remodeling (Hypertrophy and Fibrosis)

Hypertrophy and myocardial fibrosis are the most common forms of pathogenic remodeling in DCM. Therefore, the accumulation of ECM proteins, and particularly collagens, frequently characterizes the diabetic heart (26).

The pathogenesis of cardiac fibrosis in DCM is complex, involving TGF-β, the renin-angiotensin-aldosterone system (RAAS), endothelin (ET), NO, vascular growth factor (VGF), Ca_2_^+^, and tissue inhibitors of metalloproteinases (TIMPs) (27). The endothelial-to-mesenchymal transition (EndMT) is also believed to be a significant mechanism of cardiac fibrosis in diabetes. This involves the gradual acquisition of a fibroblastic phenotype and the gradual loss of the original phenotype of the ECs, and a high concentration of glucose is known to induce EndMT (28). This phenotypic shift is associated with a gradual loss of EC function, whereas the mesenchymal cell properties of the cells, such as ECM protein secretion, become more marked. Ultimately, EndMT-derived cells may function as fibroblasts in damaged tissues, but are not fully mature (28). The TGF-β, Rho/ROCK, and Notch pathways are well-known upstream regulators of EndMT. However, the factors that promote EndMT and cardiac fibrosis during the process of DCM require further study (29).

It has been reported that AGEs bind to RAGEs on various cell types, including ECs, macrophages, and smooth muscle cells, thereby activating the NF-κB signaling pathway and leading to the generation of ROS, resulting in eNOS uncoupling and lower NO availability, causing microvascular complications. AGEs crosslink ECM proteins, which, along with fibrosis, impairs myocardial relaxation. Moreover, this prevents the degradation of ECM by MMPs, further increasing myocardial stiffness. In addition, AGEs can promote the differentiation of fibroblasts into myofibroblasts, which can secrete matrix proteins. The increase in the concentration of AGEs causes the upregulation of profibrotic signals, such as angiotensin II (Ang II) and TGF-β, and the consequent imbalance between MMPs and TIMPs also promotes cardiac fibrosis. In the diabetic heart, OS also causes an increase in the intracellular Ca^2+^ concentration and a reduction in sarcoplasmic Ca^2+^ uptake, which are responsible for myocyte hypertrophy and also promote myocardial fibrosis (30). Finally, a high glucose concentration causes activation of the RAAS, leading to an increase in Ang II concentration, vascular resistance, and aldosterone secretion, which causes cardiomyocyte hypertrophy, hypertension, and the proliferation of cardiac fibroblasts (27).

### 2.6 Cardiac Dysfunction

Metabolic disorders, including hyperglycemia, IR, hyperinsulinemia, and dyslipidemia, result in OS, inflammation, accumulation of AGEs, damage to mitochondria, unbalanced calcium regulation, and cardiomyocyte apoptosis. These promote cardiac fibrosis and ultimately result in the development of diastolic or systolic dysfunction (27).

## 3 Oxidative Stress- Related Pathways

### 3.1 Glucose and Lipid Metabolism-Related Signaling

#### 3.1.1 PI3K/AKT Signaling Pathway

**Figure 2:**
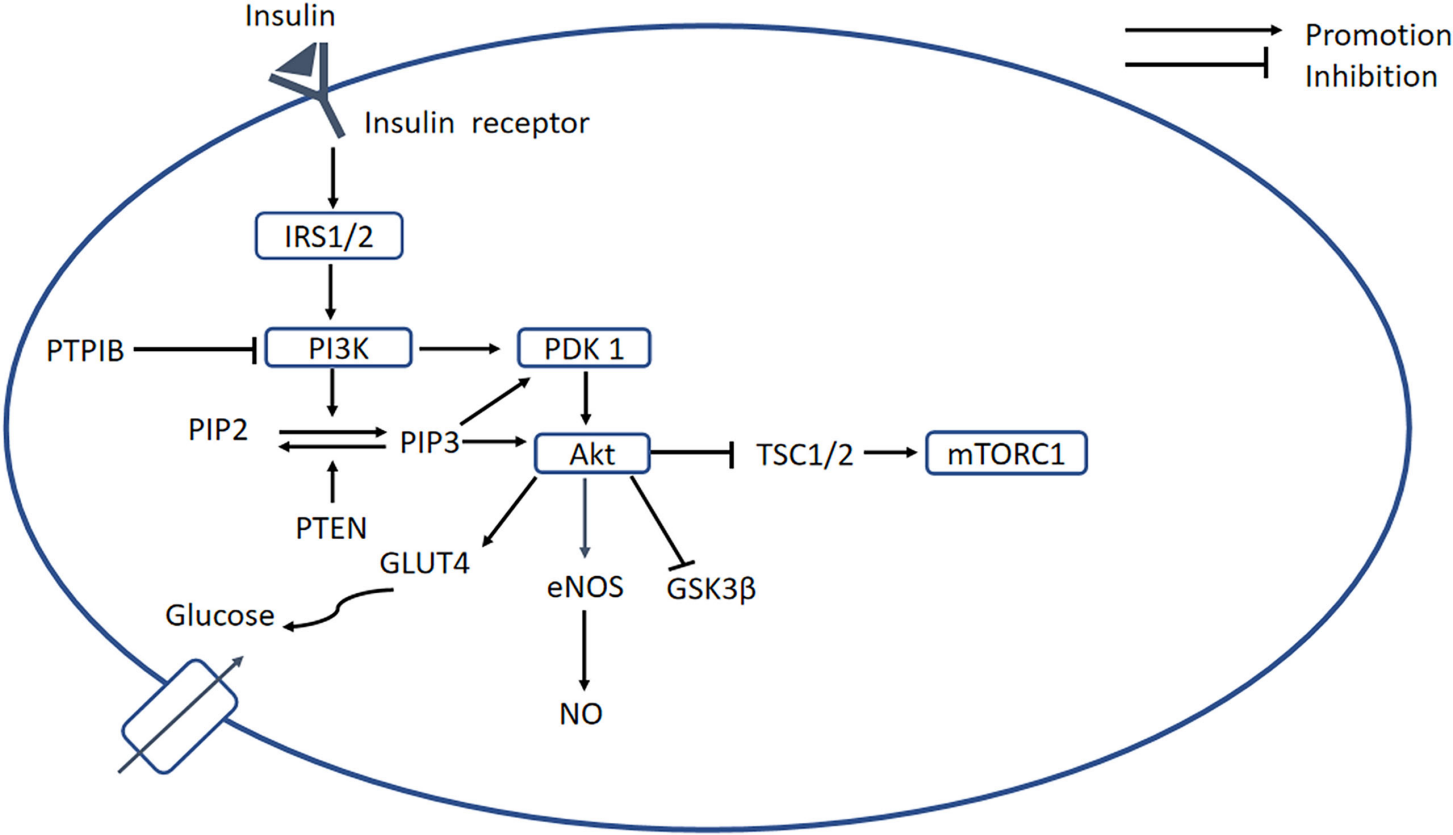
PI3K/AKT signaling pathway. Insulin binds to its receptor, causing the recruitment of downstream signaling molecules, such as IRSs. The phosphorylation of IRS enables binding of PI3K, which phosphorylates PIP2 to form PIP3, while PTEN can dephosphorylate PIP3. A high concentration of PIP3 causes the recruitment of PDK1, which promotes activation of AKT. In turn, AKT induces glycogen storage by inhibiting GSK-3β, and increases uptake of glucose by promoting translocation of GLUT4 vesicles. PTP1B serves as an important negative regulator of insulin signaling. In addition, AKT inhibits mTORC1 by inhibiting TSC1/2, and PI3K/AKT activates eNOS, leading to the release of NO. In DCM, the PI3K/AKT pathway is blocked, resulting in decreases in intracellular glucose transport and NO bioavailability. PI3K, phosphatidylinositol 3-kinase; PKB/AKT, protein kinase B; IRSs, insulin receptor substrates; PIP2, phosphatidylinositol 4;5-bisphosphate; PIP3, phosphatidylinositol 3;4;5-bisphosphate; PDK1, 3-phosphoinositide-dependent protein kinase 1; mTORC1, mechanistic target of rapamycin complex 1; TSC1;2, tuberous sclerosis complex 1;2; GSK-3β, glycogen synthase kinase 3β.

The PI3K-AKT signaling pathway is an important regulator of glucose metabolism and protein synthesis, and it is activated by insulin binding to its receptor (31). The tissue’s ability to respond to insulin is greatly reduced, which is defined as IR, and this is a key defect in T2DM (31). In DCM, IR is attributed to ROS impairment of insulin signaling (32). As shown in **Figure 2**, the binding of insulin to its receptor induces several changes, which permit the recruitment of downstream insulin receptor substrates (IRSs). Tyrosine phosphorylation of IRSs enables the binding of PI3K, which phosphorylates phosphatidylinositol 4,5-bisphosphate (PIP2) to form phosphatidylinositol 3,4,5-trisphosphate (PIP3) at the plasma membrane. Phosphatase and tensin homolog deleted on chromosome ten (PTEN) is encoded by a tumor suppressor gene, and the PTEN protein has a dual-specific phosphatase activity that inhibits activation of the PI3K/AKT pathway. Therefore, high PTEN expression can induce IR (33). Increased PIP3 causes the recruitment of 3-phosphoinositide-dependent protein kinase 1 (PDK1) and protein kinase B (PKB/AKT). Phosphorylated PDK1 activates AKT, which plays an important role in mediating the effects of insulin signaling. The activation of AKT promotes glycogen storage by inhibiting glycogen synthase kinase 3β (GSK-3β) and promotes the uptake of glucose by inducing the translocation of GLUT4-containing vesicles to the plasma membrane. Protein tyrosine phosphatase 1B (PTP1B) has been shown to dephosphorylate PI3K and AKT, and therefore represents a significant inhibitor of insulin signaling (31, 34).

In DCM, chronic defects in glucose and lipid metabolism result in OS, which blocks the activation of the PI3K/AKT signaling pathway, resulting in less intracellular glucose transport, which further impairs glucose and lipid metabolism. Downregulation of PI3K accelerates the development of DCM and increased PI3K(P110α) activity reduces OS, attenuating cardiac remodeling (35). Decreased Akt phosphorylation in diabetic hearts is accompanied by increased OS (36). OS also reduces the activation of the PI3K/AKT/eNOS signaling pathway, which reduces NO bioavailability, and this, along with the oxidation of low-density lipoprotein, causes endothelial damage (37). Downregulation of the PI3K/AKT/GSK-3β signaling pathway also indirectly promotes EndMT, thereby participating in the pathogenesis of cardiac fibrosis (38). Moreover, activation of Nrf2 by sulforaphane in H9c2 cells has been shown to be related to the Akt/GSK-3β/Fyn pathway (39). Furthermore, there is crosstalk between the PI3K/AKT and NF-κB signaling pathways, because the former causes the phosphorylation of IKKα and TP12. Therefore, the effects of OS on these keys signaling pathways require further research.

Due to the existence of signaling molecules downstream of the PI3K/Akt pathway, most experimental medications reduce OS and improve cardiac dysfunction by affecting the regulation of the downstream signaling of the PI3K/Akt pathway. For instance, Nicorandil is a NO donor that may inhibit PI3K/AKT-associated apoptosis in DCM (40). Carvacrol has been shown to protect against DCM by restoring PI3K/Akt-associated GLUT4 translocation (41). Curcumin can attenuate DCM *via* its effects on the Sirt1-FOXO1 and PI3K/AKT pathways (42). Other studies have shown that DCM can be prevented by the activation of the PI3K/AKT/GSK-3β signaling pathway by 25-OH-PPD (43); and by the inhibition of cardiac apoptosis, secondary to the activation of the PI3K/AKT/FOXO3a pathway by resveratrol (44). These results demonstrate the importance of the PI3K/Akt signaling pathway and also suggest its potential as a target for the treatment of DCM. Of note, a recent study showed that blood glucose variability can aggravate OS-induced cardiac inflammation and fibrosis by altering the Akt signaling pathway, which suggests the sensitivity to glucose of PI3K/Akt pathway (45). However, because the Akt signaling pathway has a central role in the multiple cellular responses to DCM and interacts with other signaling pathways, studies to develop specific inhibitors of PI3K/Akt signaling are facing many difficulties. Future studies should pay more attention to the targeting of specific genes related to the PI3K/akt signaling pathway.

#### 3.1.2 PPARα Pathway

**Figure 3:**
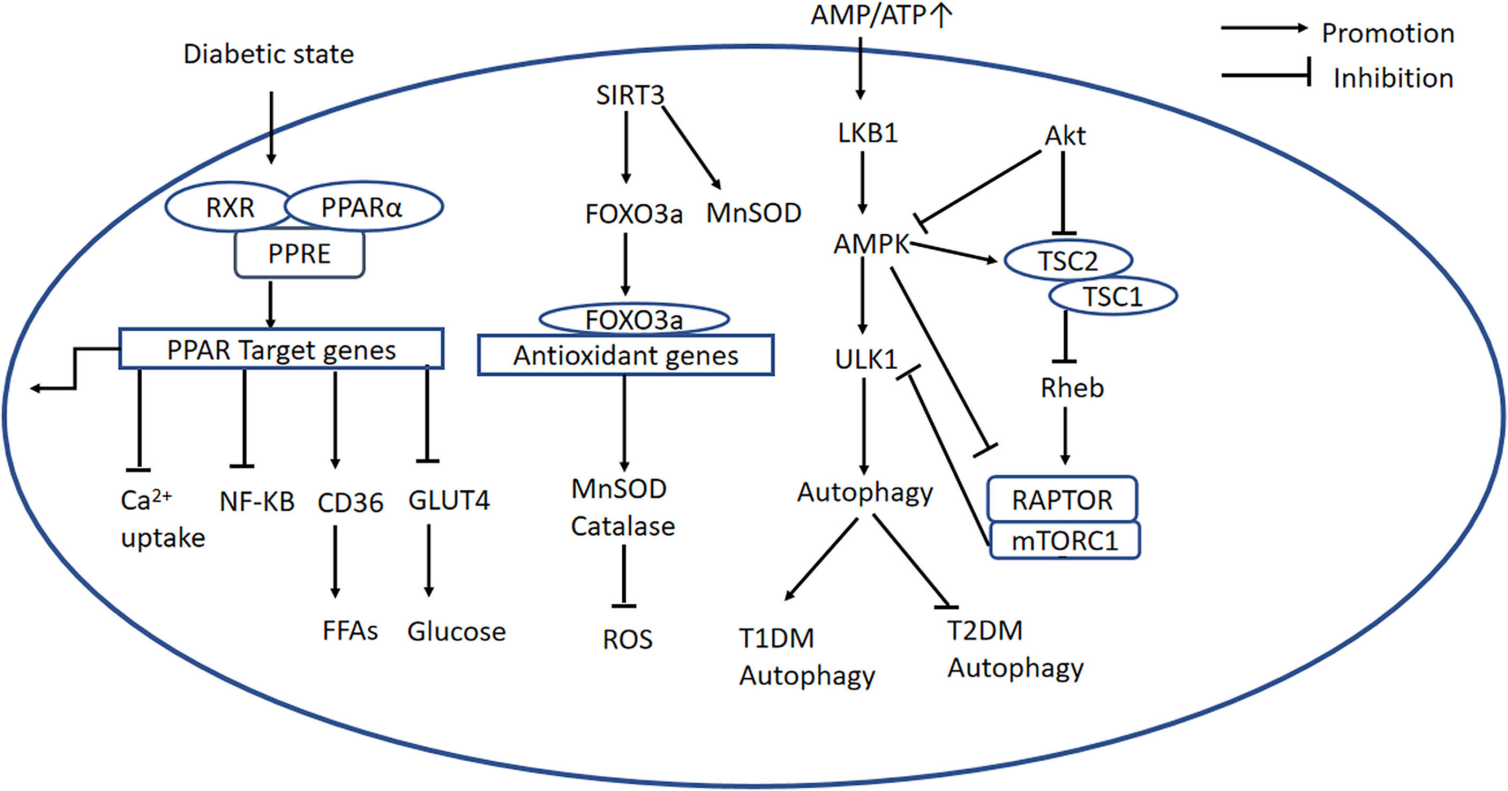
PPARα-related, AMPK/mTOR, and SIRT3/FOXO3a signaling pathways. *PPARα-related signaling pathway:* The PPARα complex is activated in the diabetic state. PPARα binds to PPREs with its heterodimeric partner RXR, regulating the expression of target genes. This upregulates CD36, inhibits GLUT4 translocation, inhibits the NF-κB signaling pathway, and inhibits Ca^2+^ uptake. *AMPK/mTOR signaling pathway:* In the diabetic heart, mitochondrial dysfunction causes a low AMP/ATP ratio, activation of LKB1, and consequent activation of AMPK. AMPK suppresses mTORC1 activation *via* two pathways. Firstly, AMPK directly phosphorylates and inhibits Raptor; secondly, it activates TSC2, and TSC2 forms a complex with TSC1, which inhibits the activity of Rheb, thereby blocking mTORC1 activation. PI3K/AKT signaling is important in mTORC1 activation because it recruits Rheb and inhibits autophagy by inhibiting AMPK. In addition, ULK1 is another regulator in autophagy, and mTOR inhibits ULK1 activation and autophagy. *SIRT3/FOXO3a signaling pathway:* SIRT3 regulates autophagy through several mechanisms. First, SIRT3 deacetylates MnSOD. Second, SIRT3 expression is high during cardiomyocyte stress, leading to the deacetylation of FOXO3a, which is translocated to the nucleus where it initiates the transcription of FOXO-dependent antioxidant genes *SOD* and *catalase*. PPARα, peroxisome proliferator-activated receptor alpha; PPREs, peroxisome proliferator response elements; RXR, retinoid X receptor; CD36, cluster of differentiation 36; GLUT4, glucose transporter 4; ATP, adenosine triphosphate; AMP, adenosine monophosphate; LKB1, liver kinase B1; AMPK, adenosine monophosphate-activated protein kinase; mTORC1, mechanistic target of rapamycin complex 1; TSC1,2, tuberous sclerosis complex 1,2; Rheb, Ras homolog enriched in brain; Raptor, regulatory-associated protein of mTOR; ULK1, Unc-51-like autophagy-activating kinase 1; PI3K, phosphatidylinositol 3-kinase; PKB/AKT, protein kinase B; SIRT3, Sirtuin 3; FOXO3a, forkhead box O3a; PINK1, PTEN-induced putative kinase protein 1; MFN2, mitofusin 2.

PPARα is a nuclear transcription factor that is expressed at high levels in the heart, and participates in lipid and glucose homeostasis (46), Ca^2+^ processing, inflammation, and cardiac OS (13). The activation of PPARα by ligand binding causes heterodimerization with retinoid X receptor (RXR), and the resulting PPAR/RXR heterodimer binds to specific DNA response elements (PPREs) in gene promoters, causing the recruitment of necessary cofactors to initiate gene transcription (**Figure 3**) (47). Previous studies have shown that the expression of PPARα is high in diabetic mice, and a PPARα transgenic mouse develops a phenotype similar to DCM (47, 48). Furthermore, it has been demonstrated that the DCM phenotype induced by overexpression of PPARα can be rescued by deletion of CD36 (49). However, other studies have shown that the expression of PPARα is low in DCM (50–52), which is consistent with the poor performance of failing hearts. The reason for this discrepancy may be that the animals used in the various studies were at different stages of the disease. Therefore, future studies should systematically investigate the changes in PPARα expression during progression of DCM. Furthermore, Wang et al. proposed that the transcriptional activity of PPARα is more significant than its gene or protein expression (13).

It has also been shown that peroxisome proliferator-activated receptor-γ coactivator (PGC)-1β, which is homologous to PGC-1α, is upregulated in mouse models of T2DM. PGC-1α is a master regulator of FA oxidation, and the PGC-1β/PPARα pathway has been shown to be important in the metabolism of the hearts of animals with DCM. Knockdown of PGC-1β reduces the transcriptional activity of PPARα, thereby improving cardiac metabolism and ameliorating cardiac dysfunction (53).

PPARα also negatively regulates glucose metabolism in the diabetic heart, and transgenic mice overexpressing PPARα also show obvious downregulation of GLUT4 and genes encoding glycolytic enzymes (47). PPARα also induces transcription from the gene encoding pyruvate dehydrogenase kinase 4, which reduces pyruvate dehydrogenase activity, further reducing glucose oxidation (54).

Although the expression of PPARα in DCM has yet to be definitively characterized, several studies have shown that concentrations of OS markers are high in PPARα transgenic mice (47). In addition, a significant characteristic of PPARα transgenic mice is the accumulation of lipids in the heart. In DCM, these disorders of glucose and lipid metabolism cause OS, and the overproduction of ROS not only directly induces inflammation, but also damages DNA, lipids, and proteins, and causes AGE production and O-glycosylation, which in turn activates the NF-κB signaling pathway and induces an inflammatory response. Interestingly, PPARα principally inhibits NF-κB signaling to exert anti-inflammatory effects (55). In addition, OS is promoted by mitochondrial dysfunction: a recent study showed that the downregulation of mitofusin 2 (MFN2) is a key mediator of the defective mitochondrial turnover that characterizes D2CM, while MFN2 overexpression ameliorates DCM by promoting mitochondrial fusion and improving mitochondrial function. The transcription of *Mfn2* is known to be directly regulated by PPARα, and the downregulation of MFN2 in DCM is partly attributable to low PPARα expression (56). In addition, a recent study showed that patients and mice with diabetes have high Krüppel-like factor-5 (*KLF5*) mRNA expression. The expression of KLF5, which regulates lipid metabolism, is known to be related to high expression of PPARα in T1DM (57), whereas FOXO1 is a positive transcriptional regulator of KLF5 in diabetes (58). KLF5 directly binds to the *NOX4* promoter and induces *NOX4* expression, which leads to OS. Interestingly, in this study, FOXO1 induces KLF5 expression through a PPARα-independent mechanism and PPARα expression through a KLF5-independent mechanism. However, the deletion of either FOXO1 or KLF5 in T1DM mice does not affect the expression of PPARα-target genes, and therefore, the amelioration of cardiac dysfunction in diabetic αMHC-FOXO1^−/−^ and αMHC-KLF5^−/−^ mice appears to be independent of PPARα and its target genes (58). Thus, no definitive conclusion can be drawn regarding the expression of PPARα at present. Possible explanations for these discrepancies may be the widespread expression of PPARα in the body and crosstalk with multiple other signaling pathways.

Recent studies have focused on the signaling molecules upstream and downstream of PPARα, and although most have only considered single molecules, whereas this signaling pathway shows crosstalk with many others, they have demonstrated that PPARα has a key role in DCM. For instance, high expression of myocardial MG53 characterizes DCM, and occurs secondary to the upregulation of PPARα and the impairment in insulin signaling (59). In addition, LAZ3 (an oncogene firstly discovered in B-cell lymphomas) regulates PPARα/NRF2 signaling by downregulating miR-21 (60). It has also been shown that SIRT3 is a signaling molecule downstream of PPARα (61). Finally, a study that investigated the effect of exenatide on DCM and the mechanism involved showed that the adapter protein 1 (APPL1)-cAMP-activated protein kinase (AMPK)-PPARα axis is upregulated, NF-kB pathway activation and apoptosis are reduced versus controls; the study also showed that these effects are independent of glucose control (62). Thus, although signaling downstream of PPARα is still not fully understood, it is obvious that the pathway elicits a range of effects that are in general protective in many tissues.

### 3.2 AMPK/mTOR Signaling Pathway

AMPK is a central controller in the regulation of cellular energy homeostasis. AMPK is activated by a low AMP/ATP ratio and is usually phosphorylated by the liver kinase B1 (LKB1) (63), whereupon it positively regulates pathways connected with the production of ATP while inhibiting ATP-consuming biosynthetic processes. AMPK is also activated by Ca^2+^/calmodulin-dependent protein kinase 2 (CaMKK2), in response to an increase in intracellular Ca^2+^ secondary to glucose starvation, and DNA damage, which are AMP-independent mechanisms (64). Therefore, AMPK is a potential therapeutic target for DCM.

mTOR is an atypical serine/threonine kinase that exists as mTOR complex 1 (mTORC1) and mTORC2 (65). Raptor is the key scaffolding protein responsible for the recruitment of mTOR substrates to mTORC1 (66, 67). mTORC1 is an important downstream signaling molecule that can be activated by AMPK. A number of molecules downstream of mTOR have been identified, including ribosomal protein S6 kinase (p70S6K), eukaryotic initiation factor 4E-binding protein 1, TFEB, PPARα/γ, HIF-1, PGC- 1α, and SREBP-1 (67). Indeed, there are two common pathways for AMPK/mTOR signaling transduction. Firstly, AMPK directly phosphorylates and inhibits Raptor (68). Secondly, AMPK activates tuberous sclerosis complex 2 (TSC2), a GTPase-activating protein that forms a complex with TSC1, which inhibits the activity of Ras homolog enriched in brain (Rheb) and thereby inhibits mTORC1 activation (69, 70). PI3K/Akt signaling also plays an important role in mTORC1 activation by recruiting Rheb, and this prevents autophagy by inhibiting AMPK. Therefore, in the diabetic heart, mitochondria dysfunction results in a low ATP concentration, which activates LKB1 and therefore AMPK, and suppresses mTORC1 activation *via* the two pathways described above.

Unc-51-like autophagy-activating kinase 1 (ULK1) is another signaling molecule downstream of AMPK that participates in the activation of autophagy (71). However, studies have shown that mTOR inhibits ULK1-induced autophagy, and that mTOR inhibition promotes an interaction between AMPK and ULK1 (72). ULK1 regulates mTORC1 *via* a negative feedback loop involving the phosphorylation of Raptor. The AMPK-ULK1 interaction is required for the induction of autophagy (73); therefore, ULK1 is essential for autophagy, especially under starvation conditions. **Figure 3** summarizes the regulation of the AMPK/mTOR signaling pathway.

In general, AMPK protects cells from mitochondrial dysfunction and inhibits OS. Mitochondrial ROS (mROS) have been shown to be an atypical activator of AMPK, while AMPK-deficient cells show approximately 50% higher concentrations of mROS and higher levels of senescence than control cells. Treatment with AMPK activators reduces mROS concentrations in unstressed cells, which suggests that AMPK regulates mROS production and inhibits OS. Furthermore, a study showed that PGC-1α may be downstream of AMPK in the control of mROS homeostasis (74). But this study used mouse embryonic fibroblasts rather than cardiomyocytes. However, AMPK and OS are closely linked in DCM. Sestrin 2, a highly conserved protein that is upregulated under various stress conditions and especially under diabetes-related OS, has been shown to activate AMPK and inhibit mTOR, thereby ameliorating IR (75).

In addition, metformin, an AMPK activator, has been shown to reduce the expression of proteins associated with cardiomyocyte apoptosis; markers of OS; and inflammatory markers in the heart of *db*/*db* mice. Furthermore, a combination of metformin and atorvastatin was found to be more effective than metformin monotherapy (76). Another study showed that metformin inhibits the NLRP3 pathway in diabetic mice through an AMPK/mTOR-dependent pathway. Interestingly, in this study, the activity of mitochondrial complex I was high in cardiomyocytes incubated in high-glucose medium, whereas AMPK phosphorylation was low (77). It has been shown that mitochondrial complex I is principally responsible for the production of O• in the mitochondrial electron transport chain, which seems to contrast with the finding that mROS activates AMPK. However, this may be explained by the type of DCM present. Taking these findings together, it is clear that OS is a potent inducer of inflammation, while the AMPK/mTOR signaling pathway has an anti-inflammatory effect in general. However, it has been shown that AMPK is upregulated in the type 1 diabetic heart, but downregulated in the type 2 diabetic heart, implying that autophagic flux is activated in the former and inactivated in the latter (63, 78). Considering the tissue specificity of different subtypes of AMPK, it will be necessary to study further the roles of the different subtypes, as well as the effects of different drugs on these subtypes, to obtain a reliable theoretical basis for elucidating how dysregulation of AMPK contributes to DCM.

### 3.3 SIRT3/FOXO3a Signaling Pathway

Sirtuin 3 (SIRT3) is a mitochondrial NAD^+^-dependent protein deacetylase (79) that is expressed in the mitochondria and nucleus (80). SIRT3 is closely related to the process of ATP production. A previous study showed that mitochondria isolated from SIRT3^−/−^ hearts are characterized by lower ATP synthesis (81). The possible explanations for this are as follows. Firstly, SIRT3 interacts with mitochondrial complexes I, II, III, and IV and deacetylates them, and complexes I and III are known to be responsible for 90% of ATP production. Secondly, SIRT3 directly deacetylates the mitochondrial enzyme SOD2. Thirdly, low levels of SIRT3 causes higher acetylation of cyclophilin D, leading to the opening of mitochondrial permeability transition pores (82). Finally, SIRT3 deacetylates and thereby activates various mitochondrial enzymes, including isocitrate dehydrogenase 2, resulting in higher mitochondrial NADPH and lower glutathione concentrations (83). Besides,SIRT3 regulates glucose and lipid metabolism by deacetylating several enzymes, such as pyruvate dehydrogenase and long chain 3-hydroxyacyl-CoA dehydrogenase. Therefore, targeting SIRT3 may provide a new treatment for T2DM.

SIRT3 has cardioprotective effects by reducing OS. A previous study showed that concentrations of 4-hydroxynonenal and malondialdehyde are high in the hearts of 8-week-old SIRT3^−/−^ mice, and that 4 weeks of antioxidant treatment normalizes the 4-hydroxynonenal concentration (81). In another study, the expression of MnSOD and catalase were found to be high in the hearts of SIRT3 overexpressing mice (84), while a third study showed that elabela (a novel peptide that has effects *via* the apelin receptor) may induce the inhibition of OS by SIRT3 through the deacetylation of FOXO3a, thereby preventing myocardial injury in diabetes (85). Taken together, these findings imply that SIRT3 inhibits OS in DCM. Therefore, the development of agents that stimulate the Sirt3-mediated antioxidant stress response could lead to novel treatments for T2DM.

SIRT3 also has a strong protective effect against cardiac hypertrophy, which is closely related to OS. It has been reported that the heart mass/tibial length ratio in SIRT3^−/−^ mice is high following 4 weeks of transverse aortic constriction, which induces a chronic increase in workload (81). Another study showed that SIRT3 overexpression prevents the cardiac hypertrophic response to phenylephrine and Ang II *in vitro* and *in vivo*, which is also consistent with the anticardiac hypertrophic effect of SIRT3 (84). Most importantly, a comparison of the antihypertrophic effects of FOXO3a deficiency and SIRT3 deficiency in mice showed that FOXO3a alone, in the absence of SIRT3, was not sufficient to prevent hypertrophy, and similarly that SIRT3 requires endogenous FOXO3a to exert its antihypertrophic effects (84). FOXO3a activity is affected by the accumulation of ROS through various signaling pathways and its deacetylation by SIRT3. FOXO3a binds to response elements on antioxidant genes and initiates the transcription of superoxide dismutase 2(SOD2) and catalase (CAT). Taken together, these findings imply that the SIRT3/FOXO3a signaling pathway has antihypertrophic effects. The signaling pathways involved are shown in **Figure 3**. A recent study has demonstrated that LCZ696 (ARNI) ameliorates OS and cardiac remodeling (hypertrophy and fibrosis) by regulating MnSOD through SIRT3 (86). LCZ696 is now often administered to slow the progression of HFrEF (87), but its effects in HFpEF, which is in general caused by DCM, have yet to be fully elucidated.

In addition to its antihypertrophic effect in the heart, the SIRT3/FOXO3a signaling pathway plays a vital role in the regulation of mitophagy. Studies have shown that the number of autophagosomes are clearly lower in neonatal mouse cardiomyocytes that have been incubated in high-glucose medium. These effects are reversed by the overexpression of SIRT3, but this reversal is prevented by the inhibitor of autophagy (88). In addition, SIRT3 overexpression ameliorates the high-glucose-induced reduction in parkin expression, and the increases in acetylated FOXO3a and FOXO3a (88). Collectively, these studies show that the activation of SIRT3 causes the deacetylation of FOXO3a, causing an increase in parkin expression in cardiomyocytes, which restores cardiomyocyte autophagy, relieves OS, maintains normal mitochondrial biosynthesis, and prevents cardiomyocyte apoptosis. Another study showed that Mfn2 is a substrate of PTEN-induced putative kinase protein 1 and is a mitochondrial binding partner for parkin, which induces the ubiquitination of mitochondrial proteins that target this organelle for autophagy (89). **Figure 3** also summarizes this signaling pathway.

## 4 Inflammation-Related Pathways

### 4.1 NF-κB and the NLRP3 Inflammasome

**Figure 4:**
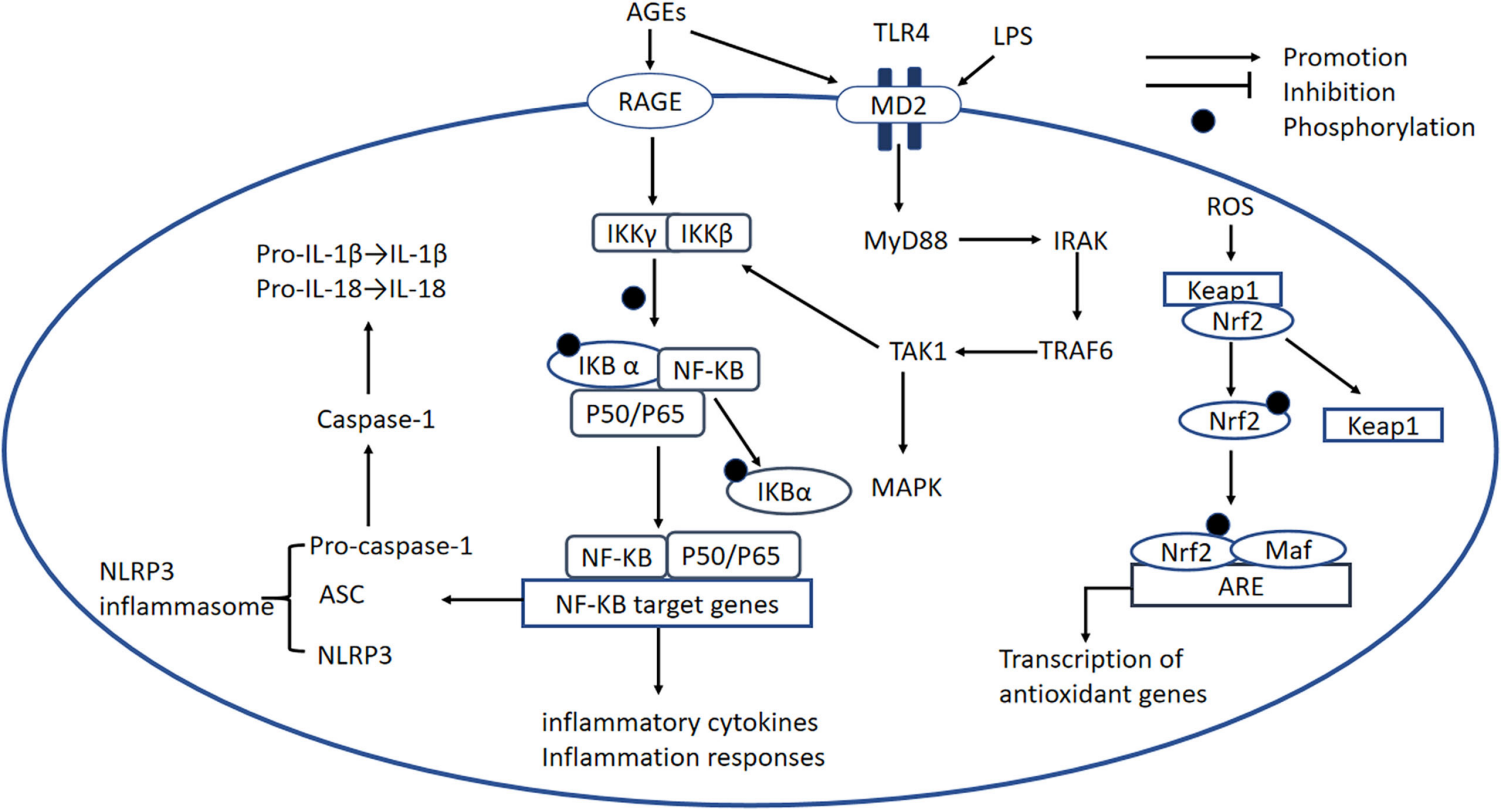
NF-κB signaling pathway, the NLRP3 inflammasome, the TLR-MyD88 signaling pathway, and the Nrf2 signaling pathway. *NF-κB signaling pathway:* In DCM, AGEs bind to RAGE, which causes the activation of RelA (p65) or RelC-containing complexes. The phosphorylation of IKKβ causes phosphorylation of IκBα and its degradation, leading to the release of NF-κB dimers. Phosphorylated NF-κB dimers bind to NF-κB DNA response elements and induce the transcription of target genes. *NLRP3 inflammasome:* The NLRP3 inflammasome complex is composed of NLRP3, ASC, and pro-caspase-1. ROS and activated NF-κB can activate the NLRP3 inflammasome. The activated inflammasome prompts ASC to cleave pro-caspase-1 to active caspase-1, which induces the maturation of IL-1β and IL-18, thereby inducing an inflammatory response. *TLR4-MyD88 signaling pathway:* AGE production is induced by OS and high glucose concentrations, and AGEs bind to MD2, leading to the formation of a TLR4/MD2 complex, which activates MyD88. In turn, MyD88 activates IRAKs and TRAF6, which activates TAK1 and the transcription factors MAPK and NF-κB. This transcription factor induces proinflammatory cytokine gene expression. *Nrf2 signaling pathway:* Nrf2 binds to its inhibitory protein Kelch-like ECH-associated protein-1 (Keap1) to form a stable complex. When it is activated by OS, Nrf2 is released and phosphorylated, and activated Nrf2 is translocated to the nucleus where it binds to antioxidant-responsive elements (AREs), initiating the transcription of antioxidant genes, including *heme oxygenase-1(HO-1)*, *NAD(P)H dehydrogenase, SOD, and catalase*. NF-κB, nuclear factor-κB; IKKβ, IkB kinase β; NLRP3, NOD-like receptor thermal protein domain-associated protein 3; ASC, apoptosis-associated speck-like protein containing a CARD; pro-caspase-1, pro-cysteinyl aspartate specific proteinase-1; MD2, myeloid differentiation-2; MyD88, myeloid differentiation primary response protein 88; IRAK, IL-1 receptor-associated kinase; TRAF6, TNF receptor-associated factor 6; TAK1, transforming growth factor-activated kinase 1; MAPK, mitogen-activated protein kinase; Nrf2, nuclear factor erythroid 2-related factor 2; Keap-1, Kelch-like ECH-associated protein-1; AREs, antioxidant-responsive elements; HO-1, heme oxygenase-1.

A large number of studies have demonstrated the importance of the NF-κB signaling pathway in the pathogenesis of DCM. There are at least two separate pathways whereby NF-κB is activated. The “canonical” pathway has been implicated in DCM: proinflammatory cytokines such as TNFα and IL-1 activate RelA (p65) or RelC-containing complexes (90). NF-κB is inactive when present in the cytoplasm, where it is bound to the inhibitory protein IκB (91), but the phosphorylation of IKKβ results in the ubiquitination of IκBα and therefore its release from NF-κB (91). The activated NF-κB is translocated into the nucleus, where it initiates inflammatory gene transcription (**Figure 4**) (92).

It has been shown that NF-κB activity is increased while gene expression of IκB is decreased in the heart of diabetic mice (23).Recent studies have shown that an IKKβ inhibitor and multiple antioxidants reduce NF-κB activation, proinflammatory cytokine secretion, and ROS concentrations in various models of diabetes (92–94). Another study showed that high expression of IκBα, which can be phosphorylated by IKKβ, also reduces NF-κB activation (95). These data show that IKKβ and IκBα may be important targets for the regulation of OS-induced inflammatory responses. In addition, a recent study showed that the levels of nuclear p65 proteins are increased in cells under high glucose conditions and another study showed that the transfection of p65 siRNA markedly suppresses the high glucose-induced upregulation of inflammatory markers (96–98). P65 proteins are localized to the cytoplasm and high glucose concentrations promote the translocation of NF-κB-P65 to the nucleus. Thus, inhibitors of IKKβ, and P65 should be evaluated further to see if they have potential for the treatment of DCM.

NF-κB and thioredoxin-interacting protein (TXNIP) have been shown to play a role in the ROS-mediated activation of caspase-1 and IL-1β, which are regulated by the NLRP3 inflammasome (99). However, a previous study of T2DM showed that TXNIP is not involved in the process of activation of IL-1β in bone marrow-derived macrophages (100). By contrast, the protein expression of activated caspase-1 and mature IL-1β is lower in TXNIP siRNA-treated or NLRP3 miRNA-treated diabetic rats than in controls (99). In addition, pharmacological inhibitors of ROS markedly reduce NF-κB phosphorylation and the expression of TXNIP, the NLRP3 inflammasome, and mature IL-1β in H9c2 cells under high glucose conditions, and pharmacological inhibitors of NF-κB downregulate the activation of the NLRP3 inflammasome (99). Taken together, these results imply that ROS and NF-κB are all activators of the NLRP3 inflammasome (**Figure 4**). The role of TXNIP seems to be controversial and reason may due to different cells selected in these studies. The activation of NLRP3 leads to the secretion of large quantities of proinflammatory cytokines, including IL-1β and IL-18, which worsen glucose intolerance and IR (101).

The NF-κB signaling pathway, its numerous upstream and downstream signaling molecules, and the crosstalk with other pathways plays an important role in DCM, but the complexity of this system necessitates further study. There have been many studies of pharmacological inhibitors of the NF-κB pathway and NLRP3 inflammasome, but more attention should be paid to assessment of the side effects of these inhibitors and their clinical translation in the future.

### 4.2 TLR4-Related Pathway

Published studies have demonstrated that the toll-like receptor 4 (TLR4) signaling pathway is a key player in the pathogenesis of DCM. Several studies have shown that hyperactivation of the TLR4 signaling pathway, which links myeloid differentiation primary response protein 88 (MyD88) with the activation of MAPK and NF-κB, and results in the expression of many proinflammatory factors, is an important mediator in DCM (102, 103). Mounting evidence shows that LPS and CD14 are key inducers of the formation of the TLR4/myeloid differentiation-2 (MD2) complex (104), which causes activation of Myd88. MyD88 recruits and activates a death domain-containing kinase, IL-1 receptor-associated kinase (IRAK) (105); another adapter protein, TNF receptor-associated factor 6(TRAF6), is downstream of IRAK (106). TRAF6 activates TGF-activated kinase 1 (TAK1), which in turn activates IκB (107). The phosphorylation of IκB promotes the nuclear translocation of NF-κB (**Figure 4**) (108). In addition, components of MAPK pathways, including c-Jun N-terminal kinase (JNK) and p38, are also activated by TAK1, leading to the production of proinflammatory cytokines (**Figure 4**) (109).

A number of studies have demonstrated that OS is associated with activation of the TLR4 signaling pathway in DCM (110, 111). Excessive ROS production in DCM activates TLR-4/MyD-88 signaling, resulting in cardiomyocyte apoptosis (110).Pharmacological inhibition of NOX in monocytes significantly reduces TLR2 and TLR4 mRNA and protein expression, and lower nuclear translocation of NF-κB is caused by pretreatment of neutrophils with the antioxidant N-acetylcysteine (111, 112). In addition, high-glucose-induced TLR2 and TLR4 expression is abolished by the p47^Phox^ siRNA treatment in monocytes (111). The inhibition of TLR4 reduces ROS concentration (113, 114) and TLR4 siRNA treatment results in the inhibition of ROS production and NOX activity in STZ-induced diabetic mice (113). Similar changes have also been identified in H9C2 cardiomyocytes (115). Collectively, these results suggest that the attenuation of OS in cardiomyocytes is associated with the suppression of TLR4.Besides,TLR4 siRNA attenuates lipid accumulation in H9C2 cardiomyocytes treated with oleic acid, implying that TLR4 may play a role in lipid metabolism (116). TLR4 seems to be a potential therapeutic target for DCM.

Other studies have shown that ROS affects NF-κB-dependent transcription by participating in early TLR4-mediated cellular responses (115). The cardiac expression of TLR4, Myd88, and NF-κB is increased in diabetic rats (117). Consistently, knockdown of TLR4 in monocytes leads to a 76% reduction in NF-κB activity under high glucose conditions (111). These data suggest that TLR4 induces inflammatory responses by activating the NF-κB-dependent pathway. MD2 expression is high in DCM, and AGEs produced in response to a high-glucose environment bind directly to MD2, leading to the activation of proinflammatory pathways. Moreover, MD2 inhibition decreases OS and inflammatory responses in diabetic rats, which in turn reduces blood pressure (114). Taken together, these data imply that MD2 deficiency protects against cardiac abnormalities in diabetes and that MD2 may be a therapeutic target for DCM (118).

To date, various pharmacological interventions have been developed that might have therapeutic potential. The H_2_S donor NaHS inactivates the TLR4/NF-κB pathway, and thereby ameliorates high-glucose-induced NLRP3 inflammasome activation and cardiotoxicity in H9c2 cells (115). In addition, lupeol, a natural triterpenoid, protects against cardiac hypertrophy by inhibiting TLR4/PI3K/AKT/NF-κB signaling (119). DCM is also attenuated by the administration of heat-inactivated *Lactobacillus reuteri* GMNL-263 to diabetic rats *via* inhibition of the TLR4 pathway (120). Furthermore, a combination of metformin and atorvastatin decreases the expression of the NLRP3 inflammasome in H9c2 cells exposed to palmitate *via* their effect on the TLR4/NF-κB signaling pathway (76). Thus, preclinical studies suggest that several pharmacological interventions may have potential for the treatment of DCM, but these findings await clinical translation.

### 4.3 Nrf2-Related Pathway

Nrf2 (nuclear factor erythroid 2-related factor 2) transcription factor functions as a key factor in redox regulation in DCM. Under physiological conditions, Nrf2 binds to its inhibitory protein Kelch-like ECH-associated protein-1 (Keap1) to form a stable complex. When it is activated by OS, Nrf2 is released and phosphorylated, and translocated to the nucleus where it binds to antioxidant-responsive elements (AREs), initiating the transcription of antioxidant genes, including *heme oxygenase-1(HO-1), NAD(P)H dehydrogenase, SOD, and catalase*. Additionally, the activation and translocation of Nrf2 can also be regulated by the PI3K/Akt signaling pathway (121).

It has been shown that Nrf2 activity and HO-1 and Keap1 expression are reduced in H9C2 cardiomyocytes treated with high glucose (122). In addition, several metabolites with antioxidant and anti-inflammatory properties, such as piceatannol and luteolin, can prevent DCM by activating Nrf2 expression (97, 98). These results indicate that Nrf2 plays an important role in antagonizing OS. It should be mentioned that the expression of Nrf2 is slightly higher in the hearts of two-month-old mice while it is lower in those of five-month-old mice (123). A possible reason for this may be adaptive overexpression of Nrf2 at the early stage of DCM while the exhausted expression induced by irreversible end-stage antioxidant system. Moreover, Nrf2 siRNA clearly increases nuclear p65 expression in cardiomyocytes under high glucose conditions (98). This study further showed that the antioxidant function of Nrf2 might be related to inhibition of the NF-κB signaling pathway. Furthermore, higher levels of antioxidant enzymes, mostly regulated by Nrf2, have been reported to prevent DCM-induced OS and cardiac hypertrophy (39, 124). Of note, Nrf2 might be a prominent actor in different cellular responses. For instance, autophagy deficiency impairs the Nrf2-driven metabolic and redox balance, which exasperates the development of DCM (125). Therefore, these results show that Nrf2 is an important factor in the pathophysiology of DCM and that its targeting could lead to a therapeutic approach for the treatment of this disease.

A large number of pre-clinical pharmacological activators of Nrf2 are natural products, including sulforaphane, curcumin, resveratrol, luteolin, metallothionein, broccoli, and garlic (97, 124, 126). Other Nrf2 activators are fumaric acid esters, carbobenzoxy-Leu-Leu-leucinal (MG132), allopurinol, and Zn (95, 122)., which upregulate Nrf2 and exert antioxidant effects that protect cardiomyocytes from OS-induced damage in DCM animal models. Both sulforaphane and luteolin are effective in protecting against inflammation, cardiac hypertrophy, fibrosis, OS, and cardiac dysfunction (97, 127). Of note, up-regulation of Nrf2 by allopurinol and MG132 may also reverse the increase in autophagy in H9C2 cardiomyocytes under high glucose conditions (122). Agonists that could potentially regulate NRF2-associated epigenetic mechanisms include methylation of the *nfe2l2* promoter and inhibitors of miR-144, miR-155, and miR-503 that upregulate NRF2 expression to attenuate cellular OS. Resveratrol prevents high glucose-induced ROS and decreases the expression of Nrf2-drived antioxidant genes by inhibiting methylation of *Nfe2l2* (128). Sulforaphane exerts its cardioprotective effect by reducing the hypermethylation of CpG islands induced by Ang-II and by promoting the accumulation of histone H3 acetylation in the Nrf2 promoter region (129). These reports show that epigenetic modification might play an important role in regulating the activation of Nrf2. Considering the key role of Nrf2/ARE signaling in antagonizing the damage caused by OS, Nrf2 is considered a potential target for the treatment of OS and OS-related diseases. Nrf2 activators will need to be further verified before use in the clinic.

## 5 Remodeling-Related Pathways

### 5.1 Rho/ROCK Signaling Pathway

**Figure 5:**
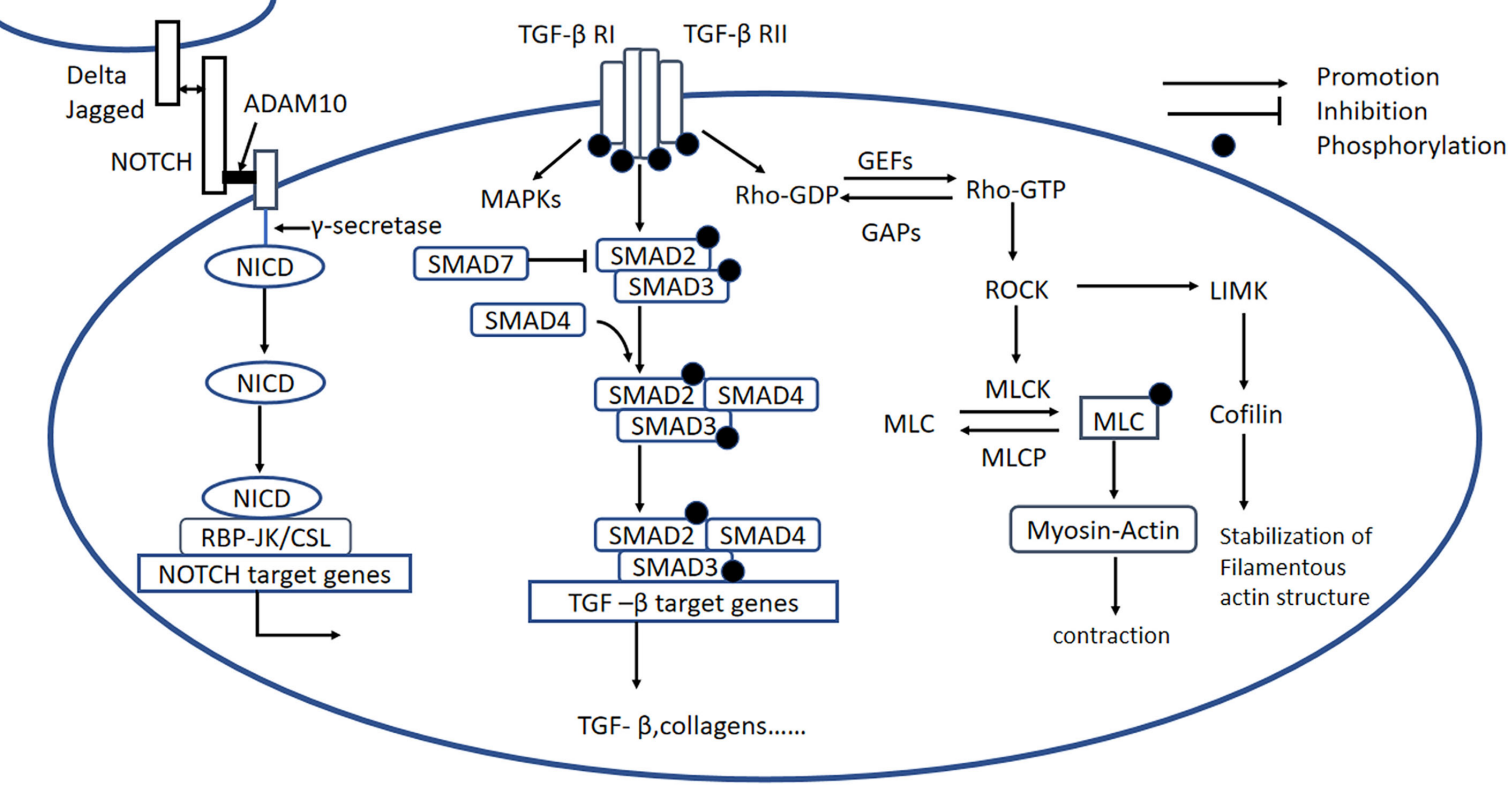
Rho/ROCK, Notch, and TGF-β signaling pathways. *Rho/ROCK signaling pathway:* Rho proteins can be activated by GEFs, involving the conversion of Rho-GDP to Rho-GTP, which activates ROCK. *Notch signaling pathway:* Notch signaling is initiated when ligands (Delta or Jagged) on the origin cell bind to Notch receptors on the recipient cell. Membrane-situated ADAM10 clamps the Notch extracellular domain to the plasma membrane and causes the release of the extracellular Notch fragment, still bound to its ligand, which initiates intracellular signaling. The remaining part of the Notch molecule is processed by a γ-secretase protease complex, which results in the release of the NICD. The NICD is then translocated to the nucleus, where it forms a Notch transcriptional activation complex with the DNA-binding protein CSL (also known as RBP-JK) and regulates Notch target gene expression. *TGF-β signaling pathway:* Active TGF-β binds to type II and type I receptors, activating downstream SMAD-dependent and SMAD-independent signaling pathways, including the RhoA and MAPK signaling pathways. GEFs, guanine nucleotide exchange factors; GAPs, GTPase-activating proteins; LIMK, LIM kinase; MLCK, myosin light chain kinase; p-MLC, phospho-myosin light chain; MLCP, phospho-myosin light chain phosphatase; ADAM10, metalloproteinase domain-containing protein; Delta/Jagged, Notch ligands; NICD, Notch intracellular domain; TGF-β, transforming growth factor β.

Rho protein is a guanylate-binding protein that exists in two states: an inactivated state, in which it is bound to GDP (GDP-Rho), and an activated state, in which it is bound to GTP (GTP-Rho). The ratio of the amount of Rho in each state is regulated by GTPase-activating proteins (GAPs) and guanine nucleotide exchange factors (GEFs). Rho kinase (ROCK) is a serine/threonine protein kinase that is the most intensively studied downstream effector of Rho (130) and exists in two known isoforms: ROCK1 and ROCK2. Although ROCK2 is more abundant in the heart and brain (131). ROCK1 is known to play an more important role in cardiovascular disease. When stimulated by histamine, thrombin, vascular endothelial growth factor, LPS, or mechanical action, Rho is activated, and the activated Rho binds to ROCK, which results in increases in calmodulin and intracellular Ca^2+^ concentrations, and the phosphorylation and activation of the myosin light chain kinase (MLCK). In addition, phosphorylation of the myosin light chain phosphatase (MLCP) inhibits the dephosphorylation of p-MLCK, and together these effects stimulate the crosslinking of myosin and actin, increasing the contraction of the actin (132). ROCK can also phosphorylate LIM kinase, which phosphorylates cofilin and thereby stabilizes actin filaments (**Figure 5**) (133).

Previous studies have shown that the RhoA/ROCK signaling pathway is activated in the hearts of STZ-induced diabetic rats, and inhibition of this pathway improves the cardiac systolic function of diabetic rats *in vitro* and *in vivo*, implying that the RhoA/ROCK pathway plays an important role in DCM (134). A high concentration of glucose activates ROCK and promotes OS, which initiates the mitochondrial death pathway, whereas the inhibition of ROCK by fasudil (a Rho/ROCK inhibitor) reduces OS and protects cardiomyocytes from OS-induced apoptosis (135). Furthermore, ROCK1-overexpressing mice are characterized by more severe cardiac fibrosis and upregulation of TGFβ1, and the administration of fasudil to rats with T2DM induced by high-fat diet-feeding in combination with STZ reduces the cardiac deposition of type I and type III collagen, and the mRNA expression of JNK and TGFβ1 (136). These findings not only show that the RhoA/ROCK signaling pathway is involved in cardiac fibrosis, but also that JNK may be downstream of the RhoA/ROCK pathway. Another study showed that OS and the phosphorylation of p38 MAPK and JNK were inhibited by fasudil in cardiomyocytes exposed to a high glucose concentration, suggesting that p38 MAPK may also be downstream of ROCK (137).

The Rho/ROCK signaling pathway also plays a role in the regulation of cardiac hypertrophy. Although under basal conditions, the hemodynamic parameters, cardiac anatomy, and cardiac function of ROCK2^−/−^mice were similar to those of wild-type (WT) mice, Ang II infusion or transverse aortic constriction reduces increases in heart mass/body mass ratios, hypertrophy-related fetal gene expression, cardiac apoptosis, and OS (138). Of note, the mice used in this study were infused with Ang II rather than having diabetes induced, but because DCM is characterized by high Ang II concentration and a similar hypertrophic phenotype, we can speculate that ROCK2 promotes cardiac hypertrophy in mice. In another study, ROCK2^+/−^ mice fed a high-fat diet showed no IR or abnormalities in left ventricular diastolic diameter, insulin signaling, or GLUT4 expression (139). These data are consistent with greater activation of ROCK2 contributing to cardiac hypertrophy and IR, and the inhibition of ROCK2, representing a potential novel target for treatment of this condition. Interestingly, it seems that ROCK1 may promote cardiac fibrosis, whereas ROCK2 may induce cardiac hypertrophy. The central role of ROCK in the relevant signaling pathways suggests that it may represent a target for the prevention or treatment of DCM, but more research is needed to ensure that inhibition of ROCK would be beneficial, and the specific roles of ROCK1 and ROCK2 should be better defined.

Fasudil is attracting considerable attention as an important ROCK inhibitor. Numerous studies have showed that fasudil can improve cardiac function, reduce myocardial impairment, and inhibit lipid peroxidation induced by OS (140). Moreover, fasudil prevents cardiac dysfunction by preserving diastolic myosin mass transfer, activating autophagy, attenuating IR, and improving calcium clearance and actin remodeling (141–144). Because of its multiple functions, fasudil is considered to be a potential treatment for DCM. However, fasudil is not currently used to treat DCM in most countries.

### 5.2 Notch Signaling Pathway

The Notch pathway regulates cell-to-cell signaling and is highly conserved. As shown in **Figure 5**, after the binding of the receptor and the ligand, the surface metalloprotease ADAM10 clamps the Notch extracellular domain to the plasma membrane; causes the release of the extracellular Notch fragment, still bound to its ligand; and induces intracellular signaling. Endopase γ-secretase cleaves the Notch intracellular domain (NICD) to release the active form of Notch into the cytoplasm, where it is translocated to the nucleus, binds with the RBP-Jκ/CSL transcription factor, which drives the transcription of target genes (**Figure 5**) (145). Notch signaling plays an important role in the development of cardiac fibrosis.

Inhibition of Notch signaling through genetic ablation of RBp-jκ has been reported to cause heart hypertrophy, which is considered to be related to impaired FFA transport, mainly mediated by Angptl4, CD36, and Fabp4, the expression of which is upregulated by Notch (146). This indicates that Notch can serve as a new target of FFA transport across the endothelium, thereby representing a promising novel therapeutic approach for the prevention of heart hypertrophy in DCM. In addition, recent studies indicated that Notch ligand Delta-like 1 (DLL1) and DLL4 play important roles in regulating glucose homeostasis. Mice lacking both DLL1 and DLL4 in adult pancreatic islet β-cells showed improved glucose tolerance, increased glucose-stimulated insulin secretion, and hyperglucagonemia, whereas DLL1 overexpression in adult β-cells had the opposite effect (147). Thus, it appears that inhibition of DLL/Notch signaling pathway partially improves β-cells insulin secretion, which is insufficient in D2CM.

Many studies have showed that the Notch1 signaling pathway is related to OS in diabetes (148–150). A recent study demonstrated that sacubitril treatment of insulin-resistant ZO rats reduces OS and Notch-1 expression in the periarterial region of the heart, suggesting that Notch1 is associated with OS. In addition, there is a significant reduction in periarterial fibrosis, but interestingly, there are no significant effects on indices of hypertrophy. Recent studies have shown that Notch 3 inhibits the differentiation of cardiac fibroblasts into myofibroblasts and the production of ECM. Importantly, fibroblast-to-myofibroblast differentiation is known to be a key process of cardiac fibrosis (151). Lentivirus-mediated Notch 3 overexpression inhibits fibroblast-to-myofibroblast differentiation in cardiac fibroblasts treated with TGF-β1 and improves cardiac fibrosis in a mouse model of myocardial infarction, which is consistent with the inhibition of cardiac fibrosis by Notch (150, 152). Moreover, downregulation of Notch2 *via* the upregulation Mia-18a-5p expression has been reported to suppress EndMT and cardiac fibrosis induced by diabetes (153). In addition, increased histone 3 lysine 4 tri-methylation in the promoter regions of Notch ligands Jagged1 and Jagged2 under intermittent high-glucose conditions has been shown to lead to the abrupt expression of these ligands and concomitant activation of Notch signaling (154). These results show that Notch-related epigenetic mechanisms may serve as potential targets for DCM treatment. However, given the wide range of cellular responses under the control of Notch signaling, the effects of targeting the Notch signaling pathway and its related molecules are likely to be complex and diverse, and many of the mechanisms have not yet been fully elucidated.

### 5.2 TGF-β Signaling Pathway

High expression of TGF-β is closely related to cardiac fibrosis induced by diabetes, hypertension, obstruction, ischemia, and toxins (155). TGF-β1 first binds to TGF-βRII to form a complex, which is beneficial for the binding of TGF-βRI. TGF-βRI is phosphorylated subsequently, resulting in the phosphorylation of SMAD proteins (156). R-SMADs (SMAD2/3) are directly activated by TGF-βRI kinase-mediated phosphorylation, whereupon they bind to SMAD4, forming a heteromeric SMAD complex. These SMAD complexes are translocated to the nucleus, where they interact with various transcriptional cofactors to regulate fibrosis-related gene expression (**Figure 5**) (157). The inhibitory SMADs (SMAD6/7) may act as a negative regulator, inhibiting the expression of TGF-β1 and relieving myocardial fibrosis. Apart from being involved in the canonical SMAD signaling pathway, TGF-βs also participate in SMAD-independent signaling pathways, including RhoA and MAPK signaling (158–160).

TGF-β is the best characterized profibrotic growth factor (161). Although the expression and activation of TGF-β have been consistently demonstrated in models of DCM, current knowledge of the role of TGF-β in fibrotic conditions has been largely derived from studies of common downstream signaling pathways. However, cardiac fibrosis represents an advanced stage of the progression of DCM. As described above, the inflammatory response induced by OS leads to production of large amounts of TGF-β by macrophages, fibroblasts, and smooth muscle cells in the injured heart, and this proinflammatory factor mediates progression of the condition.

A role for the TGF-β/SMAD signaling pathway in the development of cardiac fibrosis in DCM is supported by several lines of evidence. Firstly, several studies have shown that the overexpression and activation of TGF-β1 in DCM induce cardiac fibrosis, which can be ameliorated by administration of telmisartan, empagliflozin, dapagliflozin, or cannabidiol, probably because they inhibit TGF-β signaling (36, 162–165). However, it should be noted that the expression of downstream SMAD signaling molecules may not be affected by these interventions (162). Thus, although the TGF-β/SMAD3 signaling pathway plays an important role in DCM, the specific role of SMAD3 in DCM and the mechanisms involved remain to be fully elucidated. A recent study showed that SMAD3^−/−^ *db*/*db* mice do not exhibit cardiac dysfunction, myocardial inflammation or fibrosis, whereas SMAD3^+/−^ *db*/*db* mice are not protected against cardiac pathology, which implies that SMAD3 plays a key role in the pathogenesis of DCM (166). Moreover, SMAD3 knockdown has been shown to partially inhibit TGF-β1-induced proliferation, adhesion, and production of type I collagen in CFs. Therefore, it is possible that the noncanonical TGF-β1 pathway also contributes to the pathogenesis of cardiac fibrosis. However, given the wide range of potential mediators, the specific contribution of TGF-β to SMAD3 activation is unclear.

miRNAs have also been shown to play roles in TGF-β-induced cardiac fibrosis. Specifically, miR-195-5p downregulation has been reported to block EndMT by suppressing the TGF-β1/SMAD pathway, and the silencing of miR-195-5p inhibits EndMT through an effect on SMAD7 expression (167). In addition, the overexpression of miR-21-5p promotes EndMT, whereas inhibition of miR-21-5p suppresses TGF-β-induced EndMT. SMAD7 is reported to be the main target of miR-21-5p in endothelium (168). Moreover, inhibition of miR-150-5p improves TGF-β/SMAD2/3-induced cardiac fibrosis by increasing the level of the inhibitor protein SMAD7 (169). These results emphasize the significant role of miRNAs in the DCM process. Further work will be required to identify the specific targets of these miRNAs, and the effects of miRNAs inhibitors should be further investigated.

## 6 Pathways Regulating Ca^2+^-Dependent Contractibility

**Figure 6:**
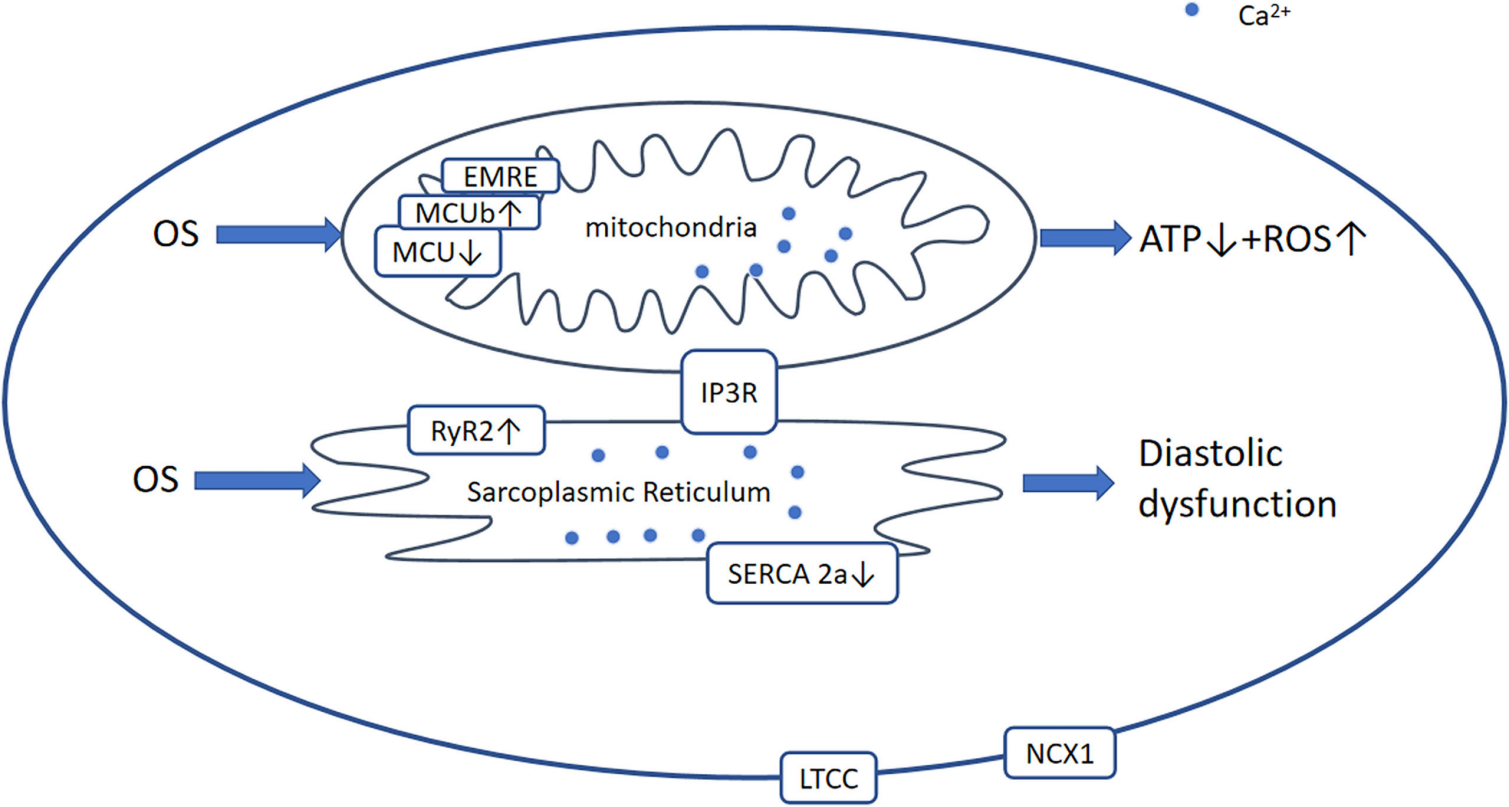
Ca^2+^-dependent signaling pathway. Generally, in DCM, OS leads to the modification and activation of RyR2 and Ca^2+^ leakage from the SR and reduced activation of SERCA2a and MCU. Therefore, [Ca^2+^]_SR_ and [Ca^2+^] _mito_ are decreased, which results in diastolic and mitochondrial dysfunction including ROS overproduction and a reduction in ATP production. OS, oxidative stress; RyR2, ryanodine receptor 2; AGEs, advanced glycation end-products; ROS, reactive oxygen species; LTCC, L‐type Ca^2+^ channel; NCX1, Na/Ca+2+-exchanger-1; MCU, mitochondrial Ca^2+^ uniporter complex; EMRE, essential mitochondrial calcium uniporter regulator; IP3R, inositol 1,4,5-triphate receptor.

Imbalanced Ca^2+^regulation is an important characteristic of both D1CM and D2CM. Although the mechanisms involved are not well understood, precise interplay among various intracellular transport protein complexes of Ca^2+^, including SR calcium-ATPase 2a (SERCA2a), L-type Ca^2+^ channel (LTCC), cardiac Na^+^/Ca^2+^-exchanger-1 (NCX1), and ryanodine receptor type 2 (RyR2), is thought to be involved (170). During the excitation-contraction coupling process, the influx of Ca^2+^ through the LTCC causes approximately two-thirds of the Ca^2+^ stored in the SR to be released *via* RyR2, thereby dramatically increasing the cytosolic Ca^2+^ concentration and inducing contraction. During diastole, approximately 95% of the cytosolic Ca^2+^ is transported back into the SR by SERCA2a, which is regulated by phospholamban (PLB). This reduces intracellular Ca^2+^ levels and promotes cardiomyocyte relaxation. The remaining Ca^2+^ largely passes through NCX1 into the extracellular milieu (170). This Ca^2+^ extrusion may contain mitochondria that are capable of storing large amounts of Ca^2+^and is important for the production of ATP (**Figure 6**) (171).

In DCM, the expression and function of SERCA2a, NCX1, LTCC, and RyR2 are affected differently by increases in the concentrations of ROS, AGEs, and proinflammatory factors. However, the effects on SERCA2a and LTCC in DCM remain to be characterized. Previous studies have shown no change, a decrease (172), or an increase in the expression of SERCA2a in *in vivo* and *in vitro* models (173–175), but the function of SERCA2a seems to be more important than its expression. Indeed, it is thought that the expression of PLB and the SERCA2a/PLB ratio are of more significance (176). However, the stimulation of SERCA2a ameliorates STZ-induced diastolic dysfunction and intracellular Ca^2+^ handling defects *in vitro* and *in vivo* (177). The expression of LTCC is considered to decrease or not to change (178, 179), and that of NCX1 is thought to be downregulated (180, 181), although some studies have suggested that greater NCX1 expression may compensate for the lower SERCA2a activity (182). In addition, RyR2 has been shown to be more active (183). Collectively, these changes delay the reuptake of Ca^2+^ by the SR and increase leakage, resulting in impaired cardiac relaxation and systolic dysfunction.

The regulation of Ca^2+^ homeostasis in mitochondria involves the voltage-dependent anion channel (VDAC), mitochondrial Ca^2+^ uniporter complex (MCU), and mitochondrial permeability transition pore (mPTP) (184). The MCU is a large complex comprising various regulatory proteins, including dominant-negative MCUb, EMRE (essential MCU regulator), and the intermembrane space regulators (EF-hand proteins) MICU1, MICU2, and MICU3. It has been shown that the hearts of diabetic mice express low levels of MCU and EMRE, and high levels of MCUb, resulting in a reduction in [Ca^2+^]*_mito_* (185). Furthermore, adenovirally induced overexpression of MCU restores mitochondrial bioenergetics (185). Another study demonstrated that low MICU1 expression is associated with OS and myocyte apoptosis in *db*/*db* mice, whereas the restoration of MICU1 expression increases [Ca^2+^]*_mito_* and inhibits the development of DCM (186). Therefore, pharmacological interventions aimed at increasing MICU1 expression or inhibiting MCUb expression may ameliorate DCM.

Ca^2+^ homeostasis is regulated by multiple transport protein complexes, proinflammatory mediators, and other factors; therefore, there are many upstream signaling molecules. A recent study showed that fasudil promotes the diastolic removal of Ca^2+^ by restoring the effect of NCX and SERCA2 (142). In addition, CXCR4 activation promotes NCX1 expression through an NF-κB-dependent pathway in cardiomyocytes in an Akitains 2 model of DCM, and this protects against systolic failure (182). Furthermore, muscle-specific SIRT3 overexpression seems to increase cardiac activation of SERCA2a in the mouse heart without deacetylating SERCA2a (187). Another study showed that the acetylation at K492, is important for SERCA2a activity (188).

## 7 Current Therapies

Currently recommended treatments for DCM patients have focused on antidiabetic, anti-inflammatory and anti-ventricular remodeling drugs. These include metformin, atorvastatin, ARNI, ACEI/ARB, sodium-glucose co-transporter 2(SGLT2), dipeptidyl peptidase-4 inhibitors (DPP-4I), glucagon-like peptide-1 receptor agonists (GLP-1RA), beta blockers, thiazolidinediones, insulin, and fasudil (189). In addition, most ongoing research has focused on natural products, suggesting that their therapeutic value merits more attention. Moreover, given that epigenetics modifications are associated with the development of DCM, the specific role of miRNAs and their inhibitors need to be further investigated.

## 8 Limitations and Perspectives

To date, OS has been considered to have a key role in the regulation of DCM. However, the precise role that OS plays in the development and progression of DCM has not been fully investigated. Most preclinical studies are aimed at clarifying the regulatory mechanisms of OS in diabetic hearts that lead to the development of DCM. However, it is unclear whether coordination occurs between OS regulation pathways in diabetic hearts. Moreover, no specific markers are available to detect the level of OS in diabetic hearts. In addition, OS inhibitors could have health safety issues. Finally, most current research on the involvement of OS in DCM has been directed toward the development of antidiabetic drugs; however, not all of these drugs have been directly observed to reduce OS parameters of the diabetic heart. At the same time, the side effects of these drugs need to be further explored. More clinical trials are needed to close the gap between preclinical findings and clinical outcomes, especially in patients with preclinical signs of DCM.

## 9 Conclusions

Preclinical studies have provided substantial evidence that a number of signaling pathways are affected by OS, which plays a significant role in the pathogenesis of DCM. OS causes abnormalities in glucose and lipid metabolism through effects on the PPARα, AMPK/mTOR, and SIRT3/FOXO3a signaling pathways. Furthermore, it is associated with inflammation mediated by the NF-κB pathway, NLRP3 inflammasome, and the TLR4 pathway. Indeed, OS and inflammation interact with each other, resulting in high concentrations of ROS and proinflammatory mediators, which promote TGF-β, Rho-ROCK, and Notch-mediated cardiac remodeling. In addition, OS is involved in the regulation of calcium homeostasis, impairment of which reduces ATP production and causes ROS overproduction. Therefore, there is no doubt that OS plays an important role in DCM. However, the mechanisms *via* which OS causes DCM are complex, and further research into the targets of OS is required to uncover the precise mechanisms.

## Author Contributions

M-lP, YF, and S-sZ conceived and edited the review. M-Lp wrote and edited the manuscript. C-wW, YZ, and HR researched data for the review. All the authors have read and agreed to the published version of the manuscript.

## Funding

This work was supported by the Natural Science Foundation of Jilin Provincial Science and Technology Department (The role of BRCA1/Nrf2/MT in chronic intermittent hypoxia-induced cardiac injury and its mechanism, 20190201035JC) and the National Natural Science Foundation of China (The role and mechanism of BRCA1 in chronic intermittent hypoxia-induced cardiac injury, 3A417C903428), Jilin Provincial Science and technology Foundation (grant number 20210509003RQ) and National Natural Science Foundation of China (No.82071570 to SZ).

## Conflict of Interest

The authors declare that the research was conducted in the absence of any commercial or financial relationships that could be construed as a potential conflict of interest.

## Publisher’s Note

All claims expressed in this article are solely those of the authors and do not necessarily represent those of their affiliated organizations, or those of the publisher, the editors and the reviewers. Any product that may be evaluated in this article, or claim that may be made by its manufacturer, is not guaranteed or endorsed by the publisher.

## Glossary

DCM: diabetic cardiomyopathy
OS: oxidative stress
ROS: reactive oxygen species
RNS: reactive nitrogen species
DM: diabetes mellitus
HF: heart failure
FAs: fatty acids
T1DM/T2DM: type 1/type 2 diabetes
IR: insulin resistance
PPAR&alpha: peroxisome proliferator-activated receptor &alpha
GLUT4: glucose transporter 4
CD36: cluster of differentiation 36
UCP: uncoupling protein
AGEs: advanced glycation end-products
HBP: hexosamine biosynthesis pathway
RAGEs: receptor for AGEs
ECM: extracellular matrix
MMPs: matrix metalloproteinase
<italic>O-GlcNAc: O</italic>-linked N-acetylglucosamine
SERCA: sarcoplasmic reticulum calcium-ATPase
NOX: NADPH oxidase
XO: xanthine oxidase
MAO: monoamine oxidase
PKC: protein kinase C
NOS: NO synthase
NO: nitric oxide
ONOO<sup>&minus</sup>: peroxynitrite anion
ECs: endothelial cells
TGF-&beta: transforming growth factor &beta
NLRPs: nucleotide-binding oligomerization domain-like receptor proteins
Pro-caspase-1: pro-cysteinyl aspartate specific proteinase-1
ASC: apoptosis-associated speck-like protein
TRX: thioredoxin
TBP-2: thioredoxin binding protein-2
RAAS: renin-angiotensin-aldosterone system
ET: endothelin
VGF: vascular growth factor
TIMPs: tissue inhibitors of metalloproteinases
EndMT: endothelial-to-mesenchymal transition
Ang II: angiotensin II
HFpEF: HF with preserved ejection fraction
HFrEF: HF with reduced ejection fraction
IRSs: insulin receptor substrates
PIP2: phosphatidylinositol 4, 5-bisphosphate
PIP3: phosphatidylinositol 3, 4, 5-trisphosphate
PI3K: phosphatidylinositol 3-kinase
PTEN: phosphatase and tensin homolog deleted on chromosome ten
PDK1: 3-phosphoinositide-dependent protein kinase 1
PKB/Akt: protein kinase B
PGC-1&alpha: peroxisome proliferator-activated receptor &gamma coactivator-1&alpha
KO: knockout
GSK-3&beta: glycogen synthase kinase 3&beta
PTP1B: protein tyrosine phosphatase 1B
PPAR&alpha: peroxisome proliferator-activated receptor alpha
RXR: retinoid X receptor
PPRE: peroxisome proliferator response element
Mfn2: mitofusin2
KLF5: Kr&uumlppel-like factor-5
APPL1: adapter protein 1
AMPK: AMP-activated protein kinase
LKB1: liver kinase B1
CaMKK2: Ca<sup>2+</sup>/calmodulin-dependent protein kinase 2
mTOR: mechanistic target of rapamycin
mTORC1/2: mTOR complex 1/2
p70S6K: p70 S6 kinase
eIF4E: eukaryotic initiation factor 4E
4EBP1: 4E (eIF4E)-binding protein 1
TSC1/2: tuberous sclerosis complex 1/2
Rheb: Ras homolog enriched in brain
ULK1: Unc-51-like autophagy-activating kinase 1
mROS: mitochondrial ROS
SIRT3: sirtuin 3
FOXO3a: forkhead Box O3a
NF-&kappa B: nuclear factor &kappaB
TXNIP: thioredoxin-interacting protein
TLR4: toll-like receptor 4
MyD88: myeloid differentiation primary response protein 88
MD2: myeloid differentiation-2
IRAK: interleukin-1 receptor-associated kinase
TRAF6: tumor necrosis factor receptor-associated factor 6
TAK1: transforming growth factor-activated kinase 1
I&kappaB: inhibitory &kappaB kinase
MAPK: mitogen-activated protein kinase
JNK: c-Jun N-terminal kinase
GAPs: GTPase-activating proteins
GEFs: guanine nucleotide exchange factors
ROCK1/2: Rho kinase1/2
MLCK: myosin light chain kinase
p-MLC: phospho-myosin light chain
MLCP: phospho-myosin light chain phosphatase
WT: wild type
NICD: Notch intracellular domain
ADAM10: metalloproteinase domain-containing protein
LTCC: L-type Ca2+ channel
NCX1: Na/Ca+2+-exchanger-1
RyR2: ryanodine receptor type 2
PLB: phospholamban
VDAC: voltage-dependent anion channel
MCU: mitochondrial Ca<sup>2+</sup> uniporter complex
mPTP: mitochondrial permeability transition pore
EMRE: essential mitochondrial calcium uniporter regulator
Nrf2: nuclear factor erythroid 2-related factor 2
Keap-1: Kelch-like ECH-associated protein-1
AREs: antioxidant-responsive elements
HO-1: heme oxygenase-1
